# A unique case of left atrium posterior wall microreentrant atrial tachycardia during sinus rhythm after pulsed field ablation in a patient with mechanical mitral valve replacement: A novel case report

**DOI:** 10.1016/j.hroo.2026.03.028

**Published:** 2026-03-27

**Authors:** Kunaraj Perumalu, Germaine Loo Jie Min, Chi Keong Ching

**Affiliations:** Department of Cardiology, National Heart Centre Singapore, Singapore

**Keywords:** Microreentrant atrial tachycardia, Pulsed field ablation, Left atrium posterior wall, Mitral valve replacement, Persistent atrial fibrillation, Anisotropic effects

**Figure:**
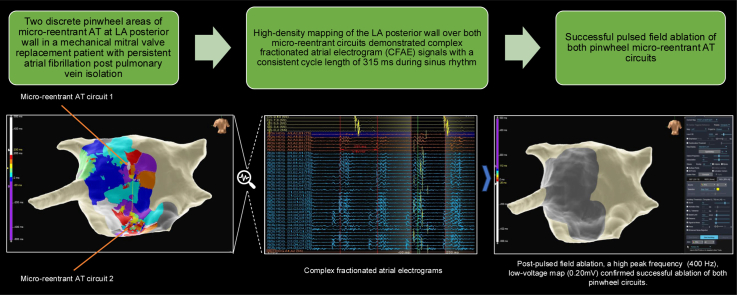

**What We Learned**

▪ High-density mapping systems play a critical role in the identification and ablation of microreentrant atrial tachycardia (AT).
▪ Microreentrant AT along the left atrial posterior wall is fundamentally linked to tissue anisotropic effects, particularly in the context of advanced atrial disease or postablation scarring.
▪ Microreentrant circuits are often small (<1 cm^2^) and easily misdiagnosed as focal tachycardias or areas of simple scarring when using conventional low-resolution mapping. Emphasis mapping is a critical tool in identifying and treating microreentrant ATs.

## Introduction

Microreentrant atrial tachycardias (ATs) originating in the left atrium (LA) posterior wall are rare. We describe a case of microreentrant AT noted while in sinus rhythm after pulsed field ablation (PFA) for atrial fibrillation (AF). Complex atrial arrhythmias require detailed 3-dimensional (3D) electroanatomic mapping with high-density (HD) mapping catheters to delineate discrete atrial circuits. There are multiple contributing mechanisms to the occurrence of microreentrant AT in the LA posterior wall.

## Case presentation

**Figure 1:**
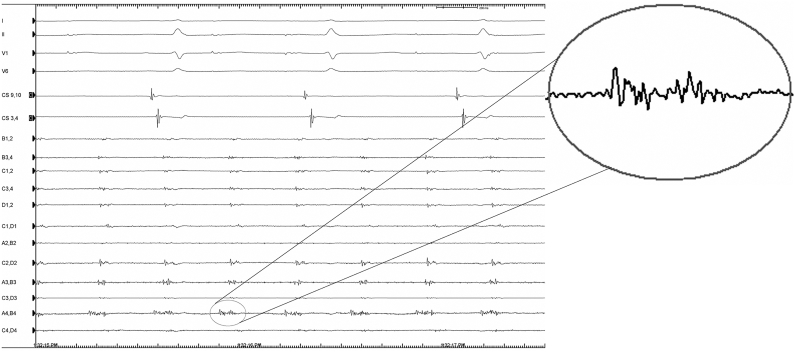
Fractionated prolonged electrogram signals of low amplitude (A4, B4) during sinus rhythm mapping with the Advisor HD Grid after pulsed field ablation in keeping with microreentrant atrial tachycardia along left atrial posterior wall.

**Figure 2:**
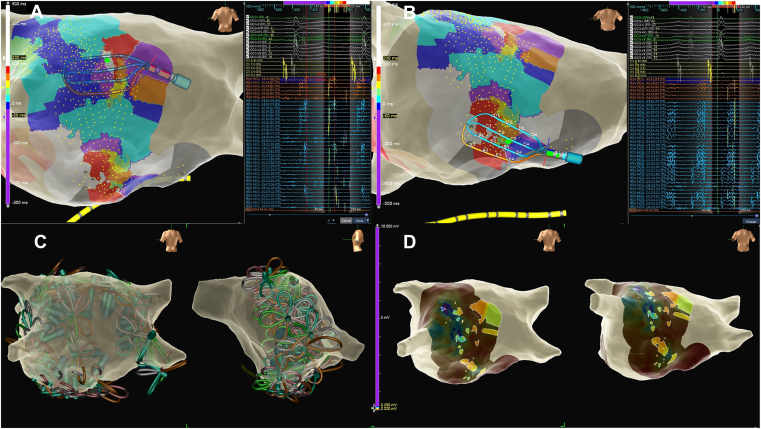
**A:** Advisor HD Grid mapping of left atrium posterior wall displaying microreentrant atrial activity during sinus rhythm. **B:** 2 discrete circuits of microreentrant atrial tachycardia with an estimated elliptical area of 56.55 mm^2^ were localized to the posterior wall of the left atrium. **C:** Post–pulsed field ablation lesion sets showed adequate coverage of all 4 pulmonary veins. **D:** Peak frequency mapping is used to identify low-voltage areas with high peak frequency (400 Hz) to delineate critical isthmuses and guide or confirm ablation targets.

A 33-year-old woman presented with symptomatic persistent AF. She had a history of congenital heart disease requiring mechanical mitral valve replacement 20 years ago. Transthoracic echocardiogram showed a preserved left ventricular ejection fraction of 60%, with a dilated LA and an LA volume index of 78.88 mL/m^2^. Her mechanical bileaflet mitral valve prosthesis function was normal with a mean transprosthetic pressure gradient of 5 mm Hg. She underwent AF ablation using the PFA system (FARAPULSE, Boston Scientific) and EnSite Precision 3D electroanatomic mapping system. An LA posterior wall microreentrant AT was observed during LA mapping in sinus rhythm after PFA with the Advisor HD Grid (Figure 1). A local activation time map showed 2 discrete circuits of microreentrant AT with the same cycle length of 315 ms (Supplemental Video 1). The 2 discrete microreentrant circuits had an estimated elliptical area of 56.55 mm^2^ along the LA posterior wall; this was further highlighted using a peak frequency emphasis map (Figure 2A and 2B). HD Grid mapping and intracardiac electrogram recording of the entire tachycardia cycle length over 2 discrete regions suggest separate focal islets of microreentry within the LA posterior wall. 2 discrete microreentrant sites were successfully treated with PFA using a flower-configuration catheter, in conjunction with LA posterior wall isolation (Figure 2C and 2D). The endocardial approach successfully terminated the microreentrant AT, with no further inducibility of AF on programmed electrical stimulation. Post-PFA HD Grid mapping showed no evidence of fractionated signals or microreentrant AT. After 5 months of follow-up, no AT/AF recurrence has been reported.

## Discussion

Microreentrant AT is attributed to a small reentrant circuit, typically defined as having a diameter of less than 2 cm, which displays localized slow conduction and a centrifugal activation pattern.^1^ The anisotropic properties of the LA posterior wall also provide a substrate for microreentrant AT, which is attributed to the heterogeneous fiber orientation leading to nonuniform anisotropy.^2^^,^^3^

The use of an HD mapping catheter with a 3D electroanatomic mapping system in complex ablation cases is beneficial because it enables accurate localization of arrhythmogenic substrate areas.^4^^,^^5^ Ultra-HD mapping in patients with persistent AF can aid in the identification of complex fractional atrial electrograms, which are a contributory factor for AF.^6^ Fragmented potentials recorded during sinus rhythm overlap significantly with actual zones of slow conduction that support microreentrant AT.

Clinical trials have shown a success rate of 90.3% at 1 year with fractionated atrial activity mapping guided ablation.^7^ In our case, the use of the Advisor HD Grid mapping catheter was invaluable for the diagnosis of the microreentrant AT.^8^ In addition, EnSite omnipolar technology with the Advisor HD Grid, which is direction independent, helped avoid bipolar blindness.^9^^,^^10^ The fractionated and prolonged electrogram patterns identified on the LA posterior wall may signify an arrhythmogenic substrate responsible for AF recurrence.^11^ Localized microreentrant circuits in the LA posterior wall are an indicator of diseased atrial substrate and a significant risk factor for the development and maintenance of AF. Moreover, the peak frequency emphasis map helped highlight the critical slow conduction areas within low-voltage areas on the LA posterior wall.^12^ In the era of PFA, these critical signals may have been completely missed if mapping was performed solely using the PFA catheter, owing to lower spatial resolution.

The occurrence of microreentrant AT in a patient in sinus rhythm is recognized as a unique feature where local reentry persists within an isolated or sequestered area of tissue.^13^^,^^14^ Slow conduction zones or partially electroporated tissue after PFA combined with preexisting surgical scar from mitral valve replacement may provide the critical isthmus required for microreentrant AT to sustain. These small circuits of microreentrant AT arise from islands of surviving myocytes within areas of incomplete irreversible electroporation.

In our case, the 2 discrete microreentrant ATs had identical cycle lengths.^15^ This can be explained by several mechanisms, including the local conduction velocity and the refractory period of the diseased tissue, both of which may play a critical role in determining the cycle length of microreentrant circuits. Microreentrant AT requires a localized zone of slow conduction and functional block, both of which are facilitated by atrial fibrosis and structural remodeling. This substrate sustains a microreentrant circuit within a small area at a consistent frequency.

Low-voltage tissues (<0.5 mV) exhibit heterogeneous conduction, which serves as a substrate for microreentrant atrial arrhythmias. In the context of complex arrhythmias such as microreentrant AT, a faster microreentrant circuit can dominate a slower one through entrainment or resetting, forcing the slower circuit to oscillate at the faster cycle length. Microreentrant ATs can be connected via the coronary sinus or vein of Marshall to the LA through striated muscle fibers (Marshall bundles) located on the epicardial side of the left lateral ridge.^16^ Marshall bundles, which are epicardial muscle bundles within the ligament of Marshall, can form arrhythmogenic connections between the LA and the coronary sinus. Microreentrant AT at the LA posterior wall is rare and has several mechanisms, as described in this case.

Finally, ablation using PFA over the LA posterior wall compared with radiofrequency ablation offers the added advantage of reduced injury risk to adjacent noncardiac structures, owing to its nonthermal profile.

## Conclusion

Microreentrant AT are rare, especially immediately after PFA. The use of advanced electrophysiology mapping technologies, incorporating HD mapping tools and 3D electroanatomic mapping software, is crucial in the identification and ablation of these arrhythmias. PFA application for microreentrant AT from the LA posterior wall is effective and safe.

## Disclosures

The authors have no conflicts of interest to disclose.

## Appendix Supplementary Data

Supplemental Video 1 A local activation map showing two discrete microreentrant AT circuits along the posterior wall of the left atrium following pulsed field ablation.
